# Enhancing gene delivery of adeno-associated viruses by cell-permeable peptides

**DOI:** 10.1038/mtm.2013.12

**Published:** 2014-02-19

**Authors:** Yarong Liu, Young Joo Kim, Man Ji, Jinxu Fang, Natnaree Siriwon, Li I Zhang, Pin Wang

**Affiliations:** 1 Mork Family Department of Chemical Engineering and Materials Science, University of Southern California, Los Angeles, California, USA; 2 Department of Physiology and Biophysics, University of Southern California, Los Angeles, California, USA; 3 Department of Biochemistry and Molecular Biology, University of Southern California, Los Angeles, California, USA; 4 Department of Biomedical Engineering, University of Southern California, Los Angeles, California, USA; 5 Department of Pharmacology and Pharmaceutical Sciences, University of Southern California, Los Angeles, California, USA

## Abstract

Adeno-associated virus type 2 (AAV2) is considered a promising gene delivery vector and has been extensively applied in several disease models; however, inefficient transduction in various cells and tissues has limited its widespread application in many areas of gene therapy. In this study, we have developed a general, but efficient, strategy to enhance viral transduction, both *in vitro* and *in vivo*, by incubating viral particles with cell-permeable peptides (CPPs). We show that CPPs increase internalization of viral particles into cells by facilitating both energy-independent and energy-dependent endocytosis. Moreover, CPPs can significantly enhance the endosomal escape process of viral particles, thus enhancing viral transduction to those cells that have exhibited very low permissiveness to AAV2 infection as a result of impaired intracellular viral processing. We also demonstrated that this approach could be applicable to other AAV serotypes. Thus, the membrane-penetrating ability of CPPs enables us to generate an efficient method for enhanced gene delivery of AAV vectors, potentially facilitating its applicability to human gene therapy.

## Introduction

Adeno-associated virus type 2 (AAV2), a relatively well-characterized AAV serotype, has been evaluated in gene therapy clinical trials owing to its ability to establish long-term gene expression in both dividing and nondividing cells without known pathological consequences of infection.^1–4^ However, the transduction of AAV2 is inefficient in a number of nonpermissive cell types,^5–8^ limiting its application in many areas of gene therapy. Two obstacles have been proposed to contribute to the inefficient infection of AAV2 in cells. First, low expression of receptors and/or coreceptors on the cell surface of nonpermissive cells prevents binding of the viral vector to the cell surface.^9–11^ Second, after binding of viral vectors to the cell surfaces and successful internalization, an impairment occurs in the multistep intracellular trafficking process of AAV2.^8^ A novel method of enhancing AAV2 infection could overcome these obstacles, thereby expanding the utility of AAV2 vectors in gene therapy.

Several approaches have been explored to improve the transduction efficiency of AAV2 in nonpermissive cell types. For example, insertion of targeting peptides into capsid proteins of vectors enables AAV2 to bind and internalize into nonpermissive cells via alternative receptors/coreceptors.^12–14^ However, this type of capsid modification could result in low production yield, reduction of vector titer, or inefficient DNA packaging.^12,15^ An alternative way to increase cell-surface interactions of viral particles may be through cell-permeable peptides (CPPs). CPPs, small poly-basic peptides, show a superior ability to penetrate cell membranes and have been used to deliver biologically active materials, such as proteins,^16^ plasmid DNAs,^17^ liposomes,^18^ and viruses,^19^ into various cell types and tissues. CPPs show promise as an alternative strategy to chemical reagents that promote endosomal membrane disruption^20,21^ because CPPs can promote the destabilization of the endosomal membrane upon acidification of the endosomal compartment without significant toxicity.^22^ For instance, arginine-rich and histidine-rich peptides have displayed the ability to disrupt the endosomal membrane via a buffering effect within the acidic endosomal environment.^23–25^ Therefore, CPPs could enhance AAV2-mediated transduction in various cell types for gene delivery to target cells and thus overcome the obstacles noted above.

In this study, we have investigated the potential of three cell-permeable peptides (Antp, TAT-HA2, and LAH4) to enhance AAV2-mediated gene delivery. We found that these CPPs significantly increase AAV2-mediated transduction into tissues and cells, including both permissive and nonpermissive cells. We also demonstrated that CPPs facilitate internalization of AAV2 via various endocytosis pathways, including the clathrin-dependent route and energy-independent pathway. Furthermore, CPPs showed a prominent ability to promote AAV2 particle escape from endosomal membranes. We found that LAH4 exhibits a superior ability to increase viral transduction via faster internalization kinetics and higher ability to penetrate endosomal membrane, as compared to TAT-HA2 and Antp. Moreover, CPPs complexed with AAV8 were shown to significantly improve AAV8-mediated gene delivery to cells and tissues. These results demonstrate a general and efficient means for enhancing viral vector-mediated gene delivery, both *in vitro* and *in vivo*, thus expanding the utility of AAV for clinical applications.

## Results

### Enhanced AAV2 transduction mediated by CPPs

**Figure 1:**
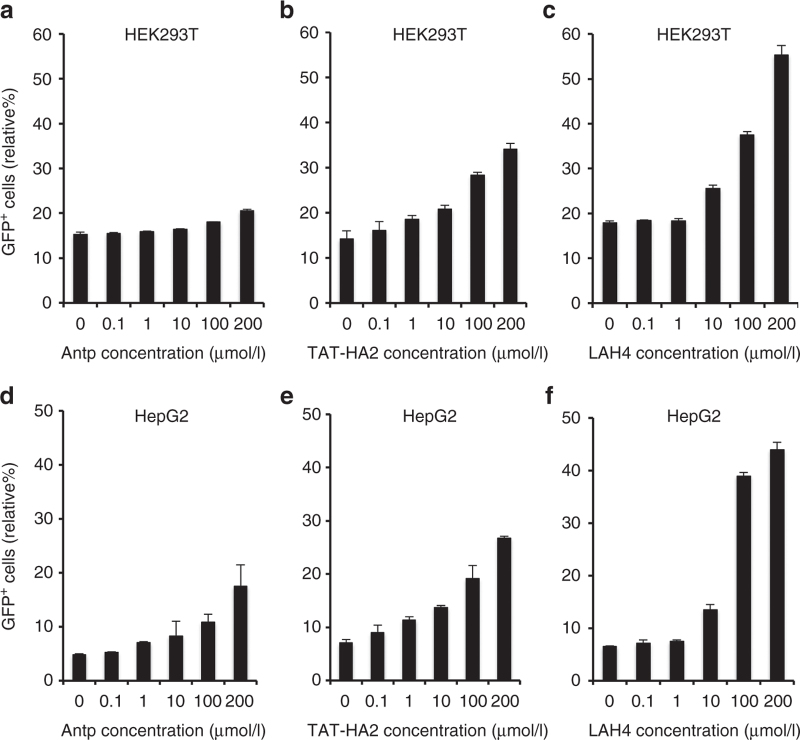
Antp, TAT-HA2, and LAH4 improve adeno-associated virus type 2 (AAV2) transduction in permissive and nonpermissive cells. (a–c) Green flourescent protein (GFP) expression levels of HEK293T cells infected with AAV2 (multiplicity of infection (MOI) = 400) alone or precomplexed with (a) Antp, (b) TAT-HA2, (c) or LAH4. (d–f) GFP expression levels of HepG2 cells infected with AAV2 (MOI = 400) alone or precomplexed with (d) Antp, (e) TAT-HA2, or (f) LAH4. Error bars represent the standard deviation of the mean from triplicate experiments.

Our main hypothesis was that preincubation of AAV2 with cell-permeable peptides (Antp, TAT-HA2, and LAH4; amino acid sequences shown in Table 1) could generally enhance transduction. To test this hypothesis, each of these CPPs was incubated with an AAV2 vector encoding green fluorescent protein (GFP), and GFP expression was assayed after infection of HEK293T cells. Increasing concentrations of CPPs (0.1–200 µmol/l) were used against fixed amounts of AAV2 (multiplicity of infection (MOI) of 400). As shown in Figure 1a–c, Antp, TAT-HA2, and LAH4 induced a dose-dependent enhancement of viral transduction in HEK293T cells. The peptide-mediated enhancement of viral transduction was further investigated in HepG2 cells, which are known to have very limited permissiveness to AAV2 infection.^7^ Indeed, flow cytometric analysis showed that transduction of AAV2 in HepG2 cells mediated by AAV2-CPPs was significantly increased from 5% up to 45% (*P* < 0.05) as compared to that mediated by AAV2 alone (Figure 1d–f). LAH4 was the most effective peptide for enhancing viral transduction in both HEK293T and HepG2 cells, as compared to that of Antp and TAT-HA2.

**Figure 2:**
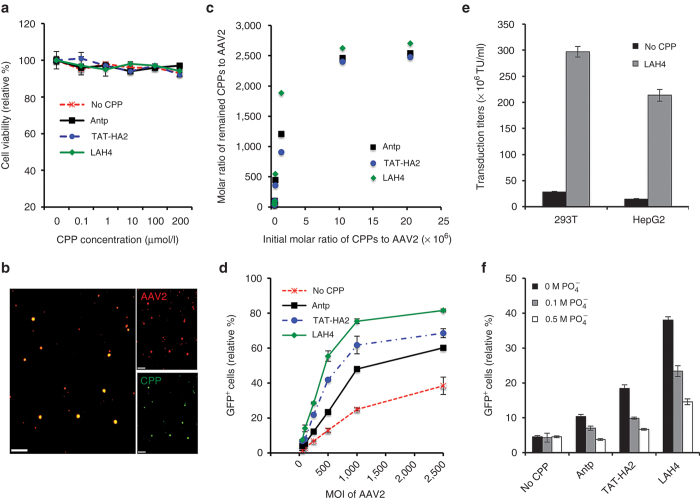
Interaction of cell-permeable peptides (CPPs) with adeno-associated virus type 2 (AAV2) facilitates enhanced viral transduction. (a) Cellular cytotoxicity of AAV2-CPP complexes. HEK293T cells were incubated with the AAV2-CPP complexes with increasing concentrations of peptides for 48 hours. Subsequently, cell viability was determined using the XTT assay. (b) Visualization of the AAV2-CPP complexes. AAV2 were incubated with FITC-CPP (green) and subsequently probed with a mouse monoclonal antibody (A20) specific for intact AAV2 (red). Overlaping green and red signals appear as yellow in the merged image. Scale bar represents 5 μm. (c) Quantitative analysis of the number of remaining CPPs per AAV2 particle. The numbers were determined by measuring the spectroscopic property of the purified AAV2-CPP complexes 30 minutes after incubation. (d) green flourescent protein (GFP) expression levels of HEK293T cells infected with increasing multiplicity of infection of AAV2 in the presence of a fixed concentration of CPPs (200 μmol/l). (e) Transduction titers of AAV2 in the presence or in the absence of LAH4 in HEK293T cells and HepG2 cells. (f) GFP expression levels of HEK293T cells infected with AAV2 alone or the AAV2-CPP complexes formed in the presence of increasing concentrations of buffered phosphate (0.1–0.5 mol/l, pH 7.4). All error bars represent the standard deviation of the mean from triplicate experiments.

To characterize the effect of these peptides on the target cells, the viability of cells was determined by an XTT assay after incubation with either AAV2-CPP complexes or AAV2 alone. As shown in Figure 2a, no cytotoxicity was observed when AAV2 was preincubated with CPPs at concentrations up to 200 μmol/l. We then investigated the complex formation between CCPs and AAV2 by confocal imaging. The purified AAV2-CPP complexes were overlaid to coverslips and immunostained with an antibody specific for intact AAV2 particles. Most of the fluorescein isothiocyanate (FITC)-labeled CPPs were colocalized with AAV2 signals (Figure 2b), confirming the formation of AAV2-CPP complexes. To further characterize the AAV2-CPP complexes, the number of CPPs functionally bound to individual AAV2 particles was determined by measuring the spectroscopic property of the purified AAV2-CPP complexes at different initial ratios as described in Materials and Methods. We found that the average maximal numbers of Antp, TAT-HA2, and LAH4 bound to each AAV2 particle are ~2,540, 2,478, and 2,706, respectively (Figure 2c).

Because we found that addition of CPPs could enhance transduction of multiple cell types, we next hypothesized that combining these CPPs with our viral vectors could reduce the amount of vectors necessary to achieve comparable levels of transduction. To test this hypothesis, we incubated constant amounts of Antp, TAT-HA2, or LAH4 (200 μmol/l) with increasing amounts of AAV2 particles and infected 293T cells at a MOI of 10−2,500. Based on the results shown in Figure 2d, the concentration of CPPs (200 μmol/l) was saturating, even for the highest amount of AAV2, with MOI of 2,500. The data in Figure 2d show that a ~3-, 10-, or 20-fold lower titer of AAV2 is sufficient for similar transduction level when AAV2 is preincubated with Antp, TAT-HA2, or LAH4, respectively. The superior potential of LAH4 on enhancing viral transduction was further confirmed by its 10- and 15-fold enhancement on viral transduction titers in HEK293T and HepG2 cells, respectively (Figure 2e).

To examine whether the positively charged nature of CPPs is critical for enhancing viral transduction via complex formation with viruses, Antp, TAT-HA2, and LAH4 (200 μmol/l) were incubated with AAV2 particles in increasing concentrations of phosphate buffer (0.1–0.5mol/l). As shown in Figure 2f, increasing concentrations of phosphate did not affect AAV2 transduction, but did significantly reduce the ability of Antp, TAT-HA2, and LAH4 to enhance viral transduction, indicating electrostatic forces in the formation of complexes between AAV2 and peptides are critical for enhancing viral transduction.

### Enhanced viral uptake with faster kinetics by CPPs

**Figure 3:**
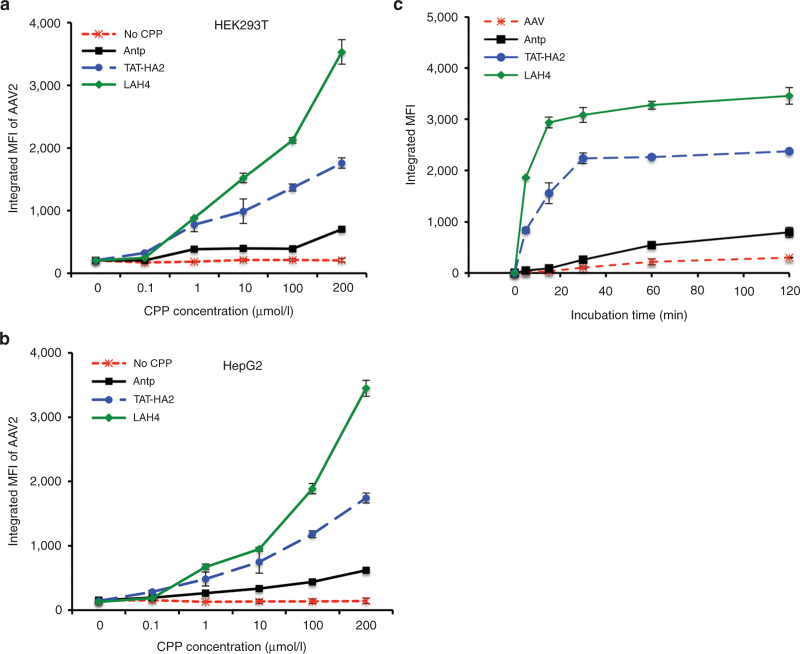
Cell-permeable peptides (CPPs) enhance adeno-associated virus type 2 (AAV2) uptake by cells. (a,b) Internalization of AAV2 particles in the presence of CPPs to (a) HEK293T cells or (b) HepG2 cells. AAV2 particles were labeled with Alexa488 dye and incubated with CPPs for 30 minutes at 37 °C. Cells were incubated with AAV2-CPP complexes for 30 minutes at 37 °C. The cellular uptake of dye-labeled AAV2 was determined by measuring dye fluorescence using flow cytometry. (c) Internalization kinetics of AAV2 particles in the presence of CPPs. AAV2 particles were labeled with Alexa488 dye and incubated with CPPs for 30 minutes at 37 °C. HEK293T cells were incubated with AAV2-CPP complexes at 37 °C for different indicated time points. After incubation, the cells were washed to remove the unbound complexes, and cellular uptake of dye-labeled AAV2 was determined by measuring dye fluorescence using flow cytometry.

We next sought to determine whether the enhanced viral transduction mediated by CPPs resulted from an increased cellular uptake of viral particles in the presence of CPPs. To measure the cellular uptake of AAV2, Alexa488-labeled AAV2 particles were preincubated with CPPs (200 μmol/l), and the uptake of viral particles was determined by flow cytometry 30 minutes after incubation with cells. As shown in Figure 3a,b, a significant increase in integrated mean fluorescence intensity of viruses was observed when cells were incubated with AAV2-CPP complexes, as compared to AAV2 alone, indicating that CPPs can facilitate the uptake of viral particles (*P* < 0.01). LAH4 showed the most prominent enhancement of AAV2 uptake as compared to TAT-HA2 and LAH4 (*P* < 0.01). Indeed, LAH4 increased cellular uptake of AAV2 at a much faster rate compared to TAT-HA2 and Antp, as shown in Figure 3c.

### Entry mechanism of AAV2-CPP complexes

**Figure 4:**
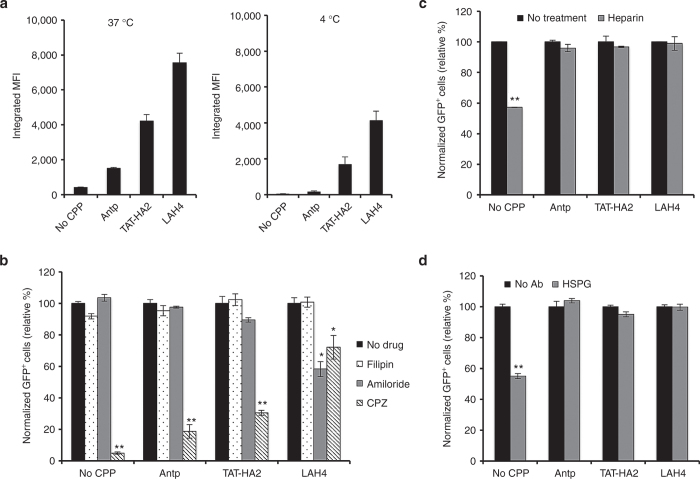
Entry mechanisms of adeno-associated virus type 2 (AAV2)-cell-permeable peptides (CPP) complexes. (a) The internalization of AAV2 or AAV2-CPP complexes in cells after 30 minutes incubation at 37 or 4 °C. (b) The effect of inhibitory drugs on viral transduction of cells infected by AAV2 or AAV2-CPP complexes. HEK293T cells were preincubated with chlorpromazine (CPZ, 30 nmol/l), filipin (15 nmol/l), or Amiloride (1 mmol/l) for 30 minutes at 37 °C. Treated and untreated cells were spin-infected with the AAV2 alone or complexes of AAV2 with peptides (final concentration: 200 μmol/l) for 90 minutes in the presence of drugs. Green flourescent protein (GFP) expression was analyzed 2 days after infection. All error bars represent the standard deviation of the mean from triplicate experiments. Asterisks indicate statistical significance compared to the no drug treatment group (*P < 0.05; **P < 0.01). (c,d) The effect of HSPG receptor blocking by (c) heparin or (d) HSPG antibody on viral transduction mediated by AAV2 or AAV2-CPP complexes. The cells were incubated with heparin (200 µg/ml) or HSPG Ab (1:100) for 30 minutes at 37 °C. The treated and untreated cells were spin-infected with the AAV2 alone or complexes of AAV2 with peptides (final concentration: 200 μmol/l) for 90 minutes. GFP expression was analyzed 2 days after infection. All data are shown as the means of triplicate experiments. Asterisks indicate statistical significance compared to the no drug treatment group (**P < 0.01).

Having shown that CPPs enhanced uptake of viral particles into the cells, we next sought to examine the mechanisms by which CPPs facilitate this viral uptake. We first investigated whether CPPs could enhance viral uptake in cells at 4 °C because AAV2 internalizes into cells primarily through clathrin-coated pits in an energy-dependent manner.^26–28^ As shown in Figure 4a, when cells were incubated with AAV2-CPP complexes at 4 °C for 30 minutes, Antp and TAT-HA2 modestly increased viral uptake compared to AAV2 alone, while LAH4 significantly enhanced the internalization of AAV2, indicating that LAH4 is the most efficient in improving energy-independent uptake of AAV2. However, the enhanced effect of CPPs on viral uptake was more apparent when AAV2-CPP complexes were incubated with cells at 37 °C, indicating that energy-dependent entry routes are also involved in the internalization of complexes.

It has been proposed that clathrin-, caveolin-, and macropinocytosis-mediated pathways are the main routes of endocytosis for viruses, proteins, and nanoparticles. To assess the role of these three endocytic pathways in the entry of AAV2-CPP complexes, drug-inhibition assays were performed. Chlorpromazine is known to block clathrin-mediated internalization,^29^ while filipin is a cholesterol-binding reagent that can inhibit the caveolin-dependent entry pathway,^30^ and amiloride is used to inhibit the macropinocytosis^31^ by preventing induction of membrane ruffling. The addition of chlorpromazine significantly decreased the transduction of AAV2-Antp, AAV2-TAT-HA2, and AAV2-LAH4 by 81.4, 69.5, and 18%, respectively (Figure 4b), indicating that the clathrin-mediated pathway is involved in endocytosis of all three AAV2-CPP complexes and that AAV2-LAH4 is the least dependent on clathrin-mediated internalization. No significant inhibitory effect of filipin was observed, suggesting that caveolin may not be involved in the entry of AAV2-CPP complexes. Moreover, only amiloride significantly decreased the transduction mediated by AAV2-LAH4 complexes, suggesting that the macropinocytosis-mediated pathway is also involved in the internalization of AAV2-LAH4 complexes, but not AAV2-Antp or AAV2-TAT-HA2 complexes.

It is generally believed that heparan sulfate proteoglycan (HSPG) is the primary attachment receptor that mediates AAV2 binding to the surface of many cell types.^32^ To assay whether HSPG is involved in the mechanism of CPP transduction enhancement, heparin and HSPG antibody were used to competitively bind to HSPG receptors.^32,33^ As shown in Figure 4c,d, both heparin and HSPG antibody treatments significantly reduced AAV2 transduction, while no inhibitory effects on viral transduction mediated by AAV2-CPP complexes were observed, indicating that CPPs facilitate viral uptake through receptors other than HSPG.

### Involvement of endosomes in viral transduction mediated by AAV2-CPP complexes

**Figure 5:**
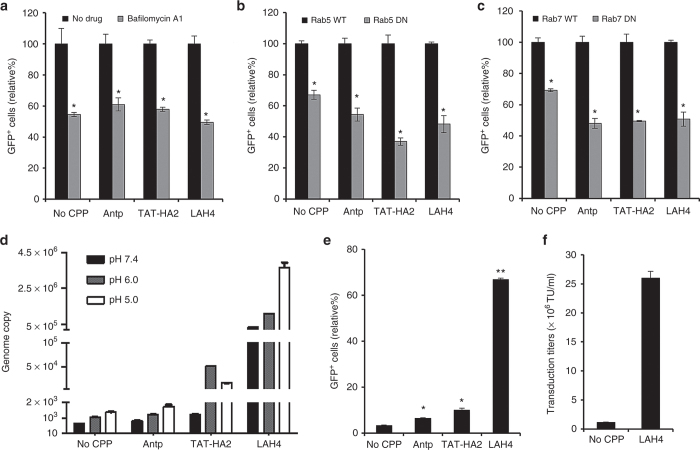
The effect of cell-permeable peptides (CPPs) on the endosomal escape of adeno-associated virus type 2 (AAV2) particles. (a) The effect of bafilomycin-A1 (BAF) on viral transduction mediated by AAV2-CPP complexes. HEK293T cells were preincubated with BAF for 30 minutes at 37 °C. The cells were then spin-infected with AAV2 or AAV2-CPP complexes for 90 minutes in the presence of BAF. GFP expression was analyzed 2 days after infection. All data are shown as the means of triplicate experiments. Asterisks indicate comparisons to the no drug treatment group (*P < 0.05). (b,c) Functional involvement of early and late endosomes in the viral transduction mediated by AAV2-CPP complexes. HEK293T cells were transiently transfected with a wild-type or dominant-negative mutant form of (b) Rab5 or (c) Rab7. Twenty-four hours after transfection, cells were spin-infected with AAV2 or AAV2-CPP complexes for 90 minutes. GFP expression was analyzed 2 days after infection. All data are shown as the means of triplicate experiments. Asterisks indicate statistical significance compared to the wild-type form of Rab treatment groups (*P < 0.05). (d) The effect of CPPs on the endosomal escape of AAV2 particles. AAV2 (10^7^ genome copies per well) or AAV2-CPP complexes (final concentration of CPP: 200 μmol/l) in phosphate-buffered saline (PBS) with different pH values (pH 7.4, 6.0, or 5.0) were placed in the upper compartment of a 24-well transwell plate. After incubation at 37 °C for 12 hours, the AAV2 particles transferred from upper compartment to the lower compartment with pH 7.4 PBS were collected. The intracellular viral genome copies were quantified by quantitative polymerase chain reaction. (e) The GFP expression of NIH3T3 cells infected by AAV2 alone or AAV2-CPP complexes (final concentration of CPP: 200 μmol/l). All error bars represent the standard deviation of the mean from triplicate experiments. Asterisks indicate statistical significance compared to the AAV2 alone treatment group (*P < 0.05; **P < 0.01). (f) Transduction titers of AAV2 in the presence or in the absence of LAH4 (final concentration: 200 μmol/l) in NIH3T3 cells. All data are shown as the means of triplicate experiments.

Endosomal transport of viral particles is thought to be a critical step for viruses to achieve successful infection. For AAV2, it has been proposed that processing through endosomal compartments, including early and late endosomes,^9,34,35^ is required to induce a conformational rearrangement of the viral capsid for nuclear transport and uncoating.^36^ To investigate whether the endosomal environments are required for productive infection of AAV2-CPP complexes, bafilomycin A1, which specifically inhibits vacuolar proton ATPases,^37^ was used to block low pH-associated endosomal processes in cells. As shown in Figure 5a, the flow cytometric analysis of GFP^+^ cells showed that transduction mediated by AAV2-CPP complexes was significantly inhibited by bafilomycin A1, indicating that low-pH endosomal processes are essential for productive infection of AAV2-CPP complexes.

To further investigate the functional involvement of different endosomes, including early and late endosomes, in AAV2-CPP-mediated transduction, the dominant-negative mutants of Rab proteins were employed to perturb either early (Rab5)^38^ or late (Rab7)^39^ endosome function. HEK293T cells transfected with wild-type or the dominant-negative form of Rab5 or Rab7 were transduced with AAV2 alone or AAV-CPP complexes. As shown in Figure 5b, expression of dominant-negative Rab5 significantly reduced the transduction rate of AAV2 and AAV2-CPPs, as compared with the transduction of wild-type Rab5-expressing cells, indicating that early endosomes are required for the AAV2-CPP infection pathway. AAV2-CPP-mediated transduction in cells expressing dominant-negative Rab7 was also remarkably decreased, compared to that of wild-type–expressing cells, suggesting that AAV2-CPP complexes traffic through both early and late endosomes for successful transduction.

It has been proposed that the increasingly acidic environment found in both early endosome (~pH 6.0) and late endosome (~pH 5.0) favors membrane penetration of AAV2 particles. Moreover, it has been reported that the acidic pH of endosomes could affect some peptide structures, such as histidine-rich molecules and poly(amido amine) polymers,^23–25^ resulting in disruption of the endosomal membrane. To investigate whether the CPPs could assist endosomal escape of AAV2 particles for enhanced transduction, we designed a membrane penetration assay using a transwell, in which a lipid bilayer membrane was formed on the bottom of the upper compartment to mimic endosomal membranes.^40^ This assay enabled us to quantify the number of viruses transiting through the membrane toward the lower compartment when either AAV2 or AAV2-CPP complexes were exposed to phosphate-buffered saline (PBS) at different pH values (7.4, 6.0, or 5.0) in the upper compartment. These pH values mimic the cytosolic, early, and late endosomal environments. As shown in Figure 5d, a moderate enhancement of virus transport was observed in AAV2-Antp complexes treated with PBS with different pH values, compared to that of AAV2 at the same pH values, suggesting that Antp could facilitate viral penetration of multiple membranes, including cell membranes and endosomal membranes. Moreover, a significant enhancement of genome copy number was observed in AAV2-TAT-HA2 complexes pretreated with buffer of pH 6.0 and 5.0, as compared to that of AAV2-Antp, indicating that TAT-HA2 could be more beneficial for endosomal escape of AAV2 in an endosomal environment. In addition, AAV2-LAH4 complexes treated with PBS with pH 7.4, 6.0, and 5.0 showed a significant movement of particles across the membrane, as compared to that of AAV2-TAT-HA2 complexes with the same treatment, suggesting that LAH4 peptide is the most efficient one in facilitating AAV2 membrane penetration, including cell membranes and endosomal membranes.

To further confirm that CPPs could facilitate endosomal escape of AAV2 particles, we examined the transduction of AAV2-CPP complexes in NIH3T3 cells, in which limited AAV2-mediated transduction has been attributed to impaired intracellular trafficking.^8,41^ As shown in Figure 5e, Antp significantly enhanced AAV2-mediated transduction in NIH3T3 cells, while TAT-HA2 induced a higher GFP expression compared to Antp. Moreover, the AAV2-LAH4 complex demonstrated the highest GFP expression in NIH3T3 cells, consistent with our previous experiments showing the superior ability of LAH4 in facilitating the endosomal escape of AAV2 particles. Indeed, we found that inclusion of AAV2-LAH4 led to a 25-fold enhancement of the transduction unit titer (~25 × 10^6^ TU/ml) in NIT3T3 cells, as compared to that of AAV2 (~1 × 10^6^ TU/ml) (Figure 5f).

### CPPs enhance viral transduction of AAV2 in primary cells and tissues

**Figure 6:**
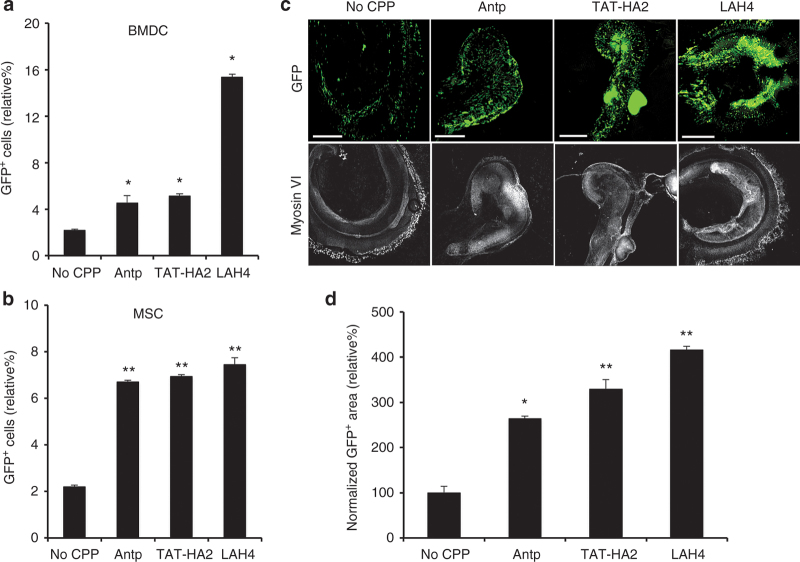
Cell-permeable peptides (CPPs) enhance viral transduction in primary cells and tissues. (a) The green flourescent protein (GFP) expression levels of bone marrow–derived cells infected by adeno-associated virus type 2 (AAV2) alone or AAV2-CPP complexes. (b) Enhancement of gene delivery of AAV2 in the presence of CPPs in murine mesenchymal stem cells. (c,d) CPPs facilitate gene delivery of AAV2 in mouse cochlear neuron. Mouse cochlear neurons were treated with AAV2-CPP complexes or AAV2 alone for 4 hours, and the medium was replaced by fresh medium. (c) After 7 days, tissues were imaged by fluorescence microscopy. Upper: GFP fluorescence. Lower: Myosin VI image. Scale bar represents 100 μm. (d) Quantification of GFP expression levels in cochlear neuron. To quantify GFP-positive cells, 4 regions of interest (ROI) were randomly chosen per image at ×10 magnification. The data are expressed as % of total area of GFP-positive in the region of AAV-CPP-treated tissues normalized by that of tissues treated by AAV2 alone. All data are shown as the means of triplicate experiments. Asterisks indicate statistical significance compared to the AAV2 alone treatment group (*P < 0.05; **P <0.01).

We have shown that cell-permeable peptides can enhance virus-mediated transduction in both permissive (HEK293) and nonpermissive (HepG2, NIH3T3) cell lines. Next, we determined whether this enhanced infection in the presence of CPPs could be achieved in primary cells. To determine this, mouse bone marrow–derived cells (BMDCs) were incubated with either AAV2 alone or AAV2-CPP complexes, and GFP expression was detected by flow cytometry 2 days after infection. As shown in Figure 6a, CPPs indeed significantly enhanced viral transduction in BMDCs, compared to AAV2 alone. As observed in the cell lines tested, LAH4 again showed a superior efficiency in increasing viral transduction compared to TAT-HA2 and Antp. Next, we tested the effect of CPPs on the viral transduction in murine primary mesenchymal stem cells (MSCs), in which AAV2-mediated transduction is inefficient.^8^ As shown in Figure 6b, the transduction efficiency mediated by AAV2-CPPs was three- to fourfold higher than that of the AAV2 alone in murine MSCs.

Additionally, we assessed the effects of the CPPs in cochlear gene transfer, which has shown promise as a potential strategy for the treatment of hearing loss.^42^ Previous studies suggested that AAV2 was unable to efficiently transduce the cells of the cochlea *in vitro*.^43^ Here, AAV2-CPP complexes displayed markedly enhanced GFP expression in cochlear tissues when compared to that of AAV2 alone (Figure 6c), as quantified in Figure 6d. It is also noteworthy that the cochlear neurons infected by AAV2-CPP complexes showed strong GFP expression in spiral ganglion cells, while gene expression was not detected in spiral ganglion cells of cochlear neurons transduced by AAV2 alone (Figure 6c). Previous research showed that no spiral ganglion cells could be transduced by a majority of serotypes of hybrid recombinant AAV vectors,^44^ demonstrating that CPPs could enhance gene transfer in tissues that cannot be achieved by alternative AAV serotypes. Taken together, this enhanced transduction mediated by AAV2-CPP complexes occurs not only in cell lines, but also in primary cells and tissues.

### CPPs enhance AAV2-mediated gene delivery *in vivo*

**Figure 7:**
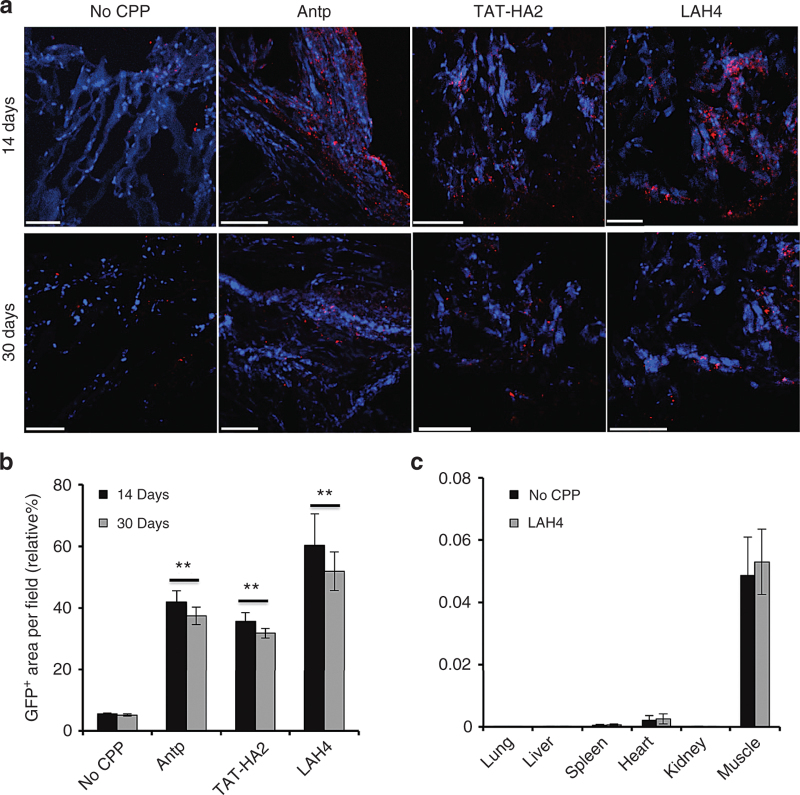
Cell-permeable peptides (CPPs) facilitate gene delivery of adeno-associated virus type 2 (AAV2) in mouse muscles. (a) Transgene expression in muscle tissues from BALB/c mice injected with AAV2 or AAV2-CPP complexes. Muscle cross sections were taken at day 14 or day 30 after intramuscular injection of AAV2 alone (10^9^ particles/mice) or AAV2-CPP complexes from injected legs. Muscle sections were stained with GFP antibody (red), followed by nuclear staining (blue). Scale bar represents 100 μm. (b) Quantification of GFP expression levels in muscles shown in (a). To quantify GFP-positive cells, four regions of interest (ROI) were randomly chosen per image at x10 magnification. The data are expressed as % of total nuclear area stained by GFP in the region. All error bars represent the standard deviation of the mean from triplicate experiments. Asterisks indicate statistical significance compared to the AAV2 alone treatment group (**P < 0.01). (c) Biodistribution of viral vectors in AAV2-injected and AAV2-LAH4-injected mice. Organs were collected from AAV2-injected and AAV2-LAH4-injected mice 14 days postadministration. AAV2 genome copies were assessed by qPCR assay. Levels of vectors were standardized using primers against housekeeping gene *Apob.* All error bars represent the standard deviation of the mean from triplicate experiments.

Recombinant AAV2 vector–based muscle gene therapy has been widely explored for inherited diseases, such as muscular dystrophies, by the ability of AAV2 vector to establish persistent transgene expression in muscles. However, it has been reported that low viral titers have limited the success of clinical trials.^45^ Many different approaches have been tested to increase the efficacy of AAV2-mediated gene delivery to muscles in order to improve the therapeutic benefit in clinical trials. Here, we investigated the potential effects of the cell-permeable peptides in an *in vivo* setting by examining gene transfer in muscle. Complexes of AAV2 (10^9^ particles) preincubated with CPPs (final concentration: 10 μmol/l) and AAV2 alone were injected intramuscularly into mice, and the GFP expression in muscles was detected 14 or 30 days postadministration. As shown in Figure 7a, intramuscular injection of AAV2 in the presence of CPPs significantly increased the GFP expression in muscles as compared to that of AAV2 alone 14 days postinjection. This enhanced gene expression via CPPs could be maintained for the duration of the 30-day study, demonstrating that the enhancement is of the long-lived nature. The potential of cell-permeable peptides on promoting *in vivo* gene delivery was further demonstrated by the quantification data shown in Figure 7b. In addition, we investigated whether CPPs could alter the biodistribution of viral vectors. As shown in Figure 7c, the vector DNA was mainly present in muscles of both AAV2-injected and AAV2-LAH4-injected mice; no significantly different distribution of viral vectors was observed between these two groups.

**Figure 8:**
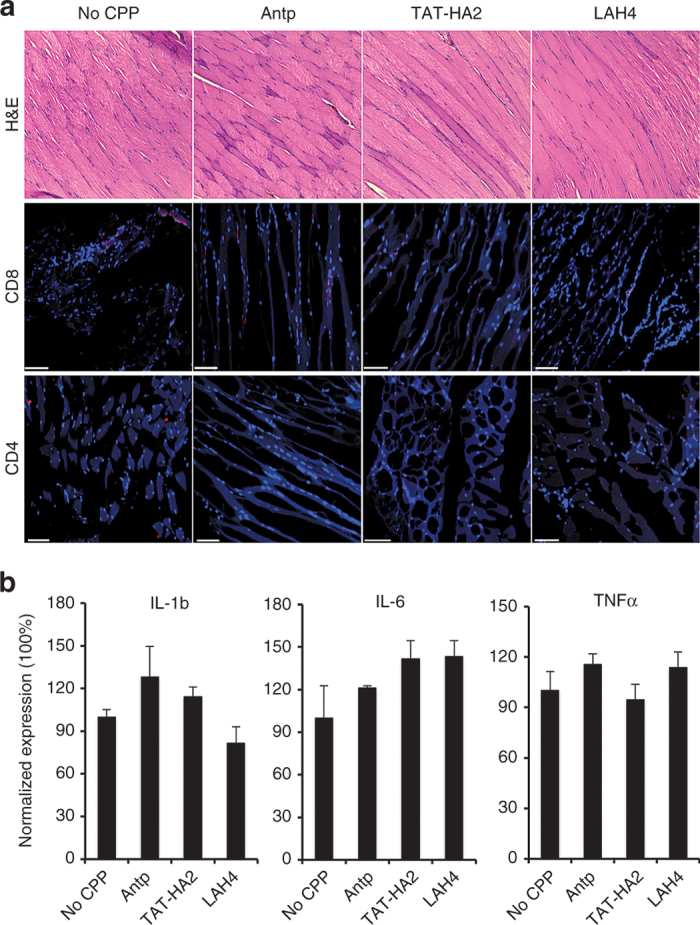
No cytotoxicity of cell-permeable peptides (CPPs) was detected *in vivo*. (a) Analysis of T lymphocyte infiltration in muscle tissues from BALB/c mice injected with adeno-associated virus type 2 (AAV2) or AAV2-CPP complexes 14 days postadministration. Serial cross-sections were stained with hematoxylin and eosin (H&E), and were immunohistochemically stained with antibodies against CD4 and CD8 (red) followed by nuclear staining (blue). Scale bar represents 100 μm. (b) Analysis of inflammation markers in muscles from mice injected with AAV2 or AAV2-CPP complexes 14 days postadministration. Total RNA was isolated from muscles and mRNA levels of interleukin (IL)-1b, IL-6, and tumor necrosis factor (TNF)-α were assessed by quantitative polymerase chain reaction. 2^Δ^Ct (^Δ^Ct = −Ct_cytokine_+Ct_GAPDH_) method was used to calculate the relative cytokine expression. All of the data were then normalized by the mean percentage of cytokine expression in AAV2-injected mice. All error bars represent the standard deviation of the mean from triplicate experiments.

Next, we determined whether CPPs could cause any cytotoxicity. As shown in Figure 8a, no significant histopathologic change was observed for the muscle tissues of the mice treated with AAV2-CPPs or with AAV2 alone. Immunohistochemical analysis revealed that AAV2-CPPs-injected muscles did not show higher rates of infiltration of CD4^+^ and CD8^+^ T lymphocytes than that of the AAV2-injected muscles, indicating that CPPs do not induce detectable cytotoxicity. To further assay for induction of innate immune gene transcripts following the intramuscular injection of AAV2-CPPs or AAV2, we performed quantitative reverse transcription polymerase chain reaction 14 days postinjection to detect the inflammatory cytokine transcript levels. Transcript levels of interleukin (IL)-1b, IL-6, and tumor necrosis factor (TNF)-α were quantified and normalized by those in AAV2-treated mice. As shown in Figure 8b, no significant difference of induced cytokine transcripts was observed in the AAV2-CPP-injected muscles as compared to that in the AAV2-injected muscles, suggesting that CPPs did not augment the host cell immune response to AAV vectors when complexed with AAV2. Taken together, CPPs were shown to significantly enhance gene delivery *in vivo* without detectable cytotoxicity.

### CPPs increase viral transduction of AAV8 both *in vitro* and *in vivo*

**Figure 9:**
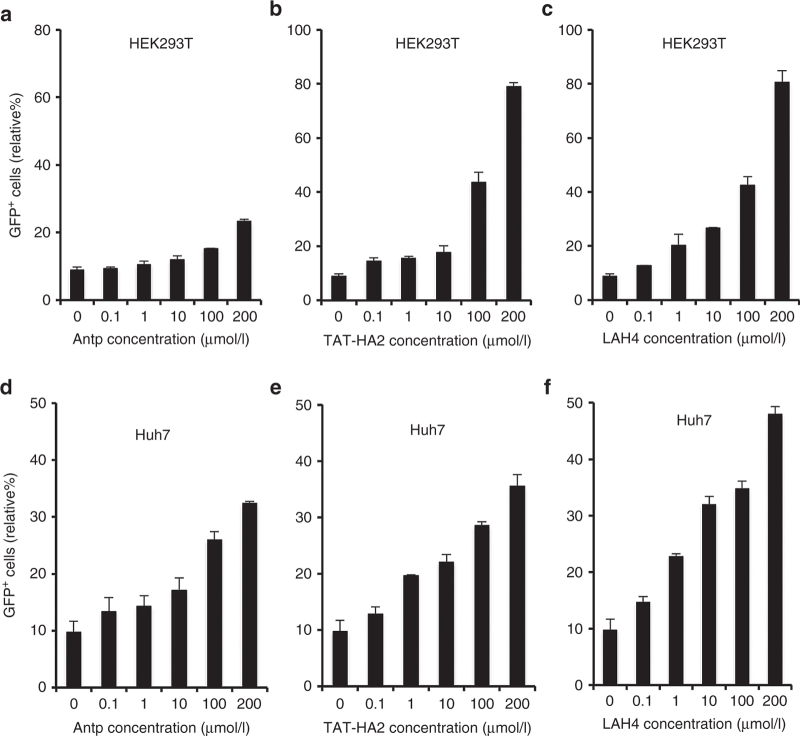
Antp, TAT-HA2, and LAH4 improve AAV8 transduction in target cells. (a–c) Green flourescent protein (GFP) expression levels in HEK293T cells infected with AAV8 (multiplicity of infection (MOI) = 1,000) alone or precomplexed with (a) Antp, (b) TAT-HA2, (c) or LAH4. (d–f) GFP expression levels in Huh7 cells infected with AAV8 (multiplicity of infection (MOI) = 1,000) alone or precomplexed with (d) Antp, (e) TAT-HA2, or (f) LAH4. Error bars represent the standard deviation of the mean from triplicate experiments.

We have showed that CPPs could significantly enhance AAV2-mediated gene delivery in various cells and tissues both *in vitro* and *in vivo*. It would be beneficial if this approach could be applicable to other serotypes, such as AAV8, which was used in clinical trials due to its unique *in vivo* transduction profiles.^46,47^ We first investigated whether CPPs could increase AAV8-mediated gene delivery in cell lines, where it shows low transduction efficiency. To test this, each of CPPs was incubated with an AAV8 vector encoding GFP, and GFP expression was assayed after transduction of HEK293T or Huh7 cells. Increasing concentrations of CPPs (0.1–200 µmol/l) were used with a fixed amount of AAV8 (MOI = 1,000). As shown in Figure 9a–c, Antp, TAT-HA2, and LAH4 induced a dose-dependent enhancement of viral transduction in HEK293T cells. In addition, transduction of AAV8 in Huh7 cells mediated by AAV8-CPPs was significantly increased as compared to that mediated by AAV8 alone (Figure 9d–f).

**Figure 10:**
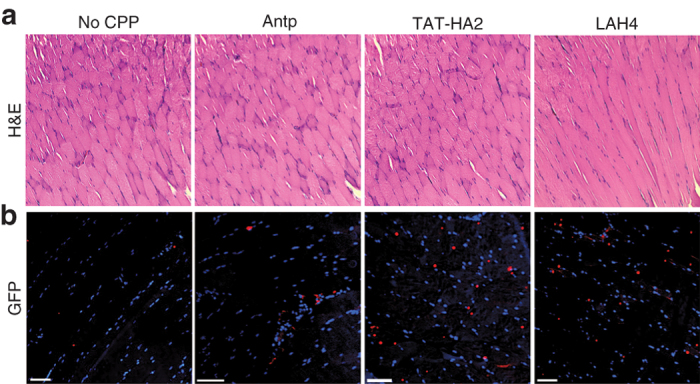
Cell-permeable peptides (CPPs) facilitate AAV8-mediated gene delivery in mouse muscles. (a,b) Histochemical and immunofluorescence analyses of muscle tissues from BALB/c mice injected with AAV8 or AAV8-CPP complexes. Muscle cross sections were taken at day 14 after intramuscular injection of AAV8 alone (10^8^ particles/mice) or AAV8-CPP complexes. Muscle sections were stained with hematoxylin and eosin (a), or GFP antibody (red), followed by nuclear staining (blue, b). Scale bar represents 100 μm.

Moreover, we investigated the potential effects of the cell-permeable peptides on AAV8-mediated gene transfer in muscles. Complexes of AAV8 (10^8^ particles) preincubated with or without CPPs (final concentration: 10 μmol/l) were injected intramuscularly into mice, and the GFP expression in muscles was analyzed 14 days postadministration. As shown in Figure 10a, no significant histopathologic change was observed for the muscle tissues from the mice treated with the AAV8-CPPs as compared to that treated with AAV8 alone, confirming that CPPs did not induce detectable cytotoxicity. More importantly, intramuscular injection of AAV8 in the presence of CPPs significantly increased the GFP expression in muscles as compared to that of AAV8 alone (Figure 10b). Thus, the fact that CPPs can enhance both AAV2 and AAV8 suggests that the approach to using CPP to improve gene delivery could be potentially applicable to various other AAV serotypes.

## Discussion

Many efforts have been made to improve the efficacy of AAV2-mediated gene transfer in cells, especially in nonpermissive cells, by increasing the internalization and endosomal escape process of viral particles. Cell-permeable peptides have been used as a promising carrier to deliver bioactive hydrophilic molecules, such as DNA, proteins, or liposomes into cells, based on their ability to penetrate cell membranes in a receptor-independent manner and to facilitate endosomal escape of molecules by disrupting endosomal membranes.^16–22^ The present investigation was designed to elucidate the role of three different CPPs (Antp, TAT-HA2, and LAH4) in AAV2-mediated gene delivery. The data presented here show that a simple preincubation of these CPP peptides with AAV2 particles markedly enhanced cellular entry and endosomal escape of AAV2 particles, thus improving both *in vitro* gene expression and *in vivo* gene delivery.

The ability of CPPs to enhance viral uptake into cells was demonstrated as a key mechanism promoting improved virus-mediated transduction of cells. Moreover, the LAH4 peptide showed a superior ability to facilitate viral uptake with a faster rate compared to Antp and TAT-HA2, even though the number of peptide molecules bound to one particle was similar. CPPs facilitate the energy-independent step of AAV2 uptake. At the same time, however, uptake requires energy-dependent internalization to achieve optimal efficiency of transduction. Although it is generally believed that AAV2 is internalized into cells via clathrin-coated pits, this study showed that LAH4 is able to facilitate the internalization of AAV2 through both clathrin- and macropinocytosis-mediated pathways. Interestingly, the competition assay suggested that CPPs facilitate the uptake of AAV2 through receptors other than HSPG. Although the detailed molecular mechanism underlying the different internalization processes of AAV-CPP complexes remains unclear, our data suggest that the differential involvement of endocytic pathways could contribute to the enhanced viral transduction in nonpermissive cell lines with limited expression of HSPG receptors and/or coreceptors.

It is generally believed that AAV2 vectors must transit through the early and late endosomes for successful infection.^7,34^ Our experiments with dominant-negative mutants of Rab constructs suggested that both early and late endosomes are required for efficient transduction of AAV2-CPPs. Moreover, viral escape from the endosomal membrane to cytosol has been considered another key barrier to viral transduction in target cells. Our previous data suggested that exposure to the acidic endosomal environment could trigger AAV2 to penetrate endosomal membranes.^40^ Here, by using an *in vitro* transwell assay, we show that CPPs could further enhance viral penetration from endosomal membranes when exposed to the acidic endosomal environment (pH 6.0 and 5.0). The ability of CPPs to facilitate endosomal escape of viral particles was further confirmed by their significant enhancement on viral transduction in NIH3T3 cells, in which previous work showed that impaired endosomal processing limited viral transduction.^8^

It has been reported that AAV2-mediated transduction of primary cells, such as BMDCs and MSCs, is inefficient. Tyrosine phosphorylation of AAV2 capsid proteins has been suggested to promote ubiquitination and degradation of the AAV2 capsid, leading to impairment of viral transport to the nucleus and thus poor transduction.^48,49^ This hypothesis was supported by the enhanced transduction of murine MSCs mediated by AAV2 tyrosine-mutant vectors.^50^ Here, we show that preincubation of AAV2 with CPPs significantly enhances viral transduction in both murine BMDCs and MSCs, offering a general and simple method that can bypass the need for manipulating AAV2 vector. Our *in vitro* transwell assay demonstrates that the addition of CPPs allows the virus to penetrate multiple types of membranes, including proteasomal membranes, and may enable viral particles to escape from the degradation process.

Alternative serotypes were usually proposed to overcome the limitations of AAV2 such as poor infectivity in certain tissues. However, certain cells such as the spiral ganglion cells in cochlear neurons are resistant to transduction by a majority of AAV serotypes.^44^ In this study, we showed that AAV2-CPP complexes could successfully transduce the spiral ganglion cells, raising the possibility that CPPs complexed with viral particles might be useful for cochlear gene therapy. We also showed that CPPs significantly enhance AAV8-mediated gene delivery both *in vitro* and *in vivo*, suggesting that this approach could be applicable to other AAV serotypes. Although it would be desirable to insert these peptides into AAV capsids, genetic incorporation of the strong cationic peptides into capsids remains challenging as it would result in a low vector production yield, dramatic reduction of vector titer, and/or significant drop of DNA packaging efficiency.^12,15^ Mixing AAVs with CPP peptides is a simple but efficient method to enhance vector transduction, sidestepping the issues of viral particle assembly, and size limit for peptide insertion.

Recombinant AAV vectors have been successfully used in several clinical trials; however, one of the biggest challenges facing AAV-based gene delivery is the host immune response. In contrast to the success of transgene delivery in mice, AAV2 induced strong immune responses, resulting in insufficient transgene expression in canine striated muscles and skeletal muscles in patients.^51,52^ This raised the concern that the dose required to produce a detectable level of gene expression was also sufficient to induce an immune response. One possible solution to this problem is to design a more efficient viral vector, which could enable gene expression at a lower vector dose. In this study, we demonstrated that CPPs could significantly enhance gene delivery in muscles without augmenting the host cell immune responses to AAV vectors. This could enable efficient vector transduction at a much lower MOI of AAVs to potentially limit the immune responses, therefore increasing the overall safety of AAV in gene delivery. In conclusion, our studies provided a general method to markedly improve AAV-mediated gene delivery to cells and tissues using cell-permeable peptides.

## Materials and Methods

### Cell lines and reagents

HEK293, HEK293T, and HepG2 cells were cultured in Dulbecco’s modified Eagle’s medium (Gibco, Big Cabin, OK) supplemented with 10% fetal bovine serum (FBS) (Sigma-Aldrich, St Louis, MO) and 2 mmol/l L-glutamine (Hyclone Laboratories, Omaha, NE). Total bone marrow cells were harvested from naive C57BL/6 mice, and BMDCs were obtained by culturing bone marrow cells in the presence of GM-CSF. Murine bone marrow–derived MSCs were isolated from 6- to 8-week-old C57BL/6 mice. Briefly, bone marrow cells were flushed out with Dulbecco’s modified Eagle’s medium supplemented with 15% FBS, centrifuged down at 1,200 rpm, and plated in a 125 cm^2^ flask. The medium was changed after 24 hours and then every 12 hours until 72 hours after collection to remove excess CD45^+^ cells. MSCs were collected after filtering out CD45^+^ cells using CD45 microbeads (MACS Miltenyi Biotech, Auburn, CA) according to the manufacturer’s protocol. Bafilomycin A1, chlorpromazine, filipin, heparin, and amiloride were obtained from Sigma-Aldrich and used at appropriate concentrations according to the manufacturer’s protocols. Anti-heparin/heparin sulfate antibody (MAB2040) was purchased from Millipore (Billerica, MA). Mouse monoclonal antibody against the intact AAV2 (A20) was purchased from American Research Products (Belmont, MA). Alexa647-conjugated goat anti-mouse IgG antibody was obtained from Invitrogen (Carlsbad, CA).

### Peptides

The Antp (amino acid sequence: RQIKIWFQNRRMKWKKC), TAT-HA2 (amino acid sequence: CRRRQRRKKRGGDIMGEWGNEIFGAIAGFLG), LAH4 (amino acid sequence: KKALLALALHHLAHLALHLALALKKAC) and FITC-labeled Antp, FITC-labeled TAT-HA2 and FITC-labeled LAH4 peptides were synthesized by GenScript (Piscataway, NJ). Peptides were dissolved in deionized water (stock solution, 10 mmol/l).

### AAV production

Recombinant AAV2 and AAV8 vectors were produced in HEK293 cells as previously described.^40^ Forty 15-cm dishes of subconfluent HEK293 cells were triple-transfected with 650 μg each of AAV cis-plasmid and AAV2 or AAV8 trans-plasmid containing the rep and cap genes and 1,300 μg of the adenovirus helper plasmid pΔF6 using the calcium phosphate precipitation method. For AAV2 production, after an additional 16 hours of incubation, the medium was replaced with fresh medium. The cells were harvested at 3 days posttransfection, followed by three cycles of freeze and thaw. For AAV8 vectors, the virus supernatants were harvested every 12 hours and replaced with fresh medium for 7 days. AAV2 or AAV8 viruses were then purified by cesium chloride gradient density centrifugation^53^ at 25,000 rpm and 15 °C for 20 hours (Optima L-90 K Ultracentrifuge, SW-28 rotor, Beckman Coulter, Brea, CA). Viral particles recovered from the first round of ultracentrifugation were pooled and subjected to isopycnic separation by a second CsCl centrifugation at 13,000 rpm and 15 °C for 20 hours in a SW-32 Ti rotor. Fractions containing AAV2 or AAV8 determined by refractive index were further desalted in PBS using an Amicon Ultra 100,000 MWCO centrifugal filter device (Millipore).

### Plasmids

The plasmid encoding the dominant-negative mutant of DsRed-Rab7 (Rab7T22N) was generated by site-directed mutagenesis as described.^54^ The constructs for wild-type and dominant-negative forms of DsRed-Rab5 and DeRed-Rab7 were obtained from Addgene (Cambridge, MA).

### Cell infection

Purified AAV2 particles (MOI of 400) or AAV8 (MOI of 1,000) were preincubated with the peptides (final concentration: 0.1–200 μmol/l) in PBS for 30 minutes at 37 °C. The AAV alone or AAV-peptide complexes were then added to the HEK293T cells, HepG2 cells, or Huh7 (2 × 10^4^ cells per well) in a 96-well culture dish by spin infection at 2,500 rpm for 90 minutes at 25 °C using a Sorvall Legend centrifuge. The medium was replaced with fresh medium after an additional 3 hours of incubation. GFP^+^ cells were detected by flow cytometry 2 days postinfection.

To assess the degree to which peptides influenced the titer necessary for expression, peptides (fixed concentration: 200 μmol/l) were preincubated with increasing MOI of AAV2. HEK293T cells were then spin-infected with the complexes for 90 minutes. GFP expression was analyzed 2 days after infection.

For viral transduction with drug-treated cells, HEK293T cells or HepG2 cells were preincubated with bafilomycin A1 (BAF, 50 nmol/l), chlorpromazine (25 µg/ml), filipin (5 µg/ml), amiloride (1 mmol/l), heparin (200 µg/ml) or HSPG Ab (1:100) for 30 minutes at 37 °C. The treated and untreated cells were spin-infected with the AAV2 alone or complexes of AAV2 with peptides (final concentration: 200 μmol/l) for 90 minutes. GFP expression was analyzed 2 days after infection.

For viral transduction with PO_4_^−^ blocking, AAV2 alone or the complexes were incubated with increasing concentrations of phosphate (0.1–0.5 mol/l) in the preincubation buffer, and the cells were then spin-infected at 2,500 rpm for 90 minutes at 30 °C using a Sorval Legend centrifuge (Newport Pagnell, England).^40^ After an additional 3 hours of incubation at 37 °C, the medium was then removed and replaced with fresh medium and cultured for 72 hours before flow cytometry analysis of GFP^+^ cells. For viral transduction with Rab protein-expressing cells, HEK293T cells were seeded in a 24-well dish overnight at 37 °C. The cells were then transfected with DsRed-Rab5 or -Rab7 (either wild-type or dominant-negative mutants) by the calcium phosphate precipitation method. After 4 hours of incubation at 37 °C, the media were then replaced with fresh D10 media (Dulbecco’s modified Eagle’s medium containing 10% FBS). At 24 hours posttransfection, the complexes of AAV2 with peptides were spin-infected with the transfected cells for 90 minutes, and the percentage of GFP-positive cells was analyzed by flow cytometry at 48 hours postinfection.

### *In vitro* cytotoxicity

HEK293T cells were plated at a density of 5 × 10^3^ cells per well in 96-well plates and grown for 6 hours. The cells were then exposed to complexes of AAV2 with different concentrations of peptides for 48 hours, and cell viability was assessed using the Cell Proliferation Kit II 2,3-bis-(2-methoxy-4-nitro-5-sulfophenyl)-2H-tetrazolium-5-carboxanilide (XTT assay) from Roche Applied Science (Indianapolis, IN) according to the manufacturer’s instructions. Cell viability percentage was determined by subtracting absorbance values obtained from media-only wells from drug-treated wells and then normalizing to the control cells without complexes.

### *In vitro* internalization study

AAV2 (1 × 10^10^ particles) was incubated with 50 nmol of Alexa488-TFP ester (Invitrogen) for 2 hours in 0.1 mol/l sodium bicarbonate buffer (pH = 9.3). After 2 hours incubation, the reaction was stopped, and unbound dye molecules were removed via buffer exchange into PBS (pH = 7.4) using a Zeba desalting spin column (Fisher Scientific, Waltham, MA). The Alexa488-labeled AAV2 were then incubated with different concentrations of peptides for 30 minutes. HEK293T or HepG2 cells were then incubated with the complexes (MOI = 400) for 30 minutes at 37 °C. After incubation, the cells were washed and treated with trypsin to eliminate unbound particles and those not uptaken. Cellular uptake of AAV particles was determined by measuring Alexa488 fluorescence using flow cytometry.

For internalization kinetics of complexes, Alexa488-labeled AAV2 was preincubated with fixed concentration of peptides (200 µmol/l) for 30 minutes. Then, the complexes were incubated with cells for different time points (5, 15, 30, 60, and 120 minutes). After incubation, the cells were washed and treated with trypsin to eliminate unbound particles and those not uptaken. Cellular uptake of AAV particles was determined by measuring Alexa488 fluorescence using flow cytometry.

### Quantification of number of peptides bound per AAV2 particle

The quantification of the number of peptides bound per AAV2 particle was determined by using the absorption measurements. First, AAV2 particles were labeled with Cy5-NHS ester (GE Healthcare, Piscataway, NJ) for 2 hours. After purification by a gel filtration column, the number of attached dyes per AAV2 particle was then calculated by measuring the absorbance of purified Cy5-labeled AAV2 particles at 650 nm for Cy5-NHS ester (ε = 250,000 M^−1^ cm^−1^) and at 280 nm for AAV2 particles (ε = 6.61 × 10^6^ M^−1^ cm^−1^).^55^ A different initial ratio of AAV2 particles to FITC-labeled peptides was incubated for 30 minutes at 37 °C. After purification by a gel filtration column, the extent of peptides on AAV2 particles was then calculated by measuring the absorbance of the purified coupling products at 650 nm for Cy5 (ε = 250,000 M^−1^ cm^−1^) and at 494 nm for FITC (ε = 68,000 M^−1^ cm^−1^).

### *In vitro* model of endosomal membrane

Twenty-four-well transwells with 0.4 μm pore filters on the bottom of the upper compartment were used to study the endosomal escape of the complexes of AAV2 with peptides. A planar lipid bilayer was formed by applying 5 μl of 1% L-α-phosphatidycholine (Sigma, St Louis, MO) on the porous filters of the upper compartment. The complexes of AAV2 (10^7^ genome copies per well) with peptides (final concentration: 200 μmol/l) in PBS with indicated pH values were incubated in the upper compartment of transwell for 12 hours. The vectors transferred to the lower compartment with pH 7.4 PBS were collected, and intracellular genomes were extracted and quantified by quantitative polymerase chain reaction assay.

### Cochlear tissue culture and infection

C57BL/6J mice were sacrificed at P0-P2 by decapitation using procedures approved by the USC Animal Committee. Individual cochlears were dissected in cold Leibovitz’s L-15 (L-15) medium and explanted onto a glass-bottom dish previously coated with CellTek (BD Biosciences, San Jose, CA). Explants were then incubated in Dulbecco’s modified Eagle’s medium/F-12 supplemented with 10% FBS, N2 supplement (Invitrogen), and penicillin at 37 °C. For viral transfection experiments, the complexes of AAV2 (10^9^ particles) with CPPs (Antp, TAT-HA2, and LAH4) and AAV2 vectors alone were applied for 4 hours directly to culture media. Tissues were placed back in culture for 7 days to allow for stable and robust expression of the transfected constructs. The culture media were exchanged daily. At the end of each experiment, cultures were fixed in 4% paraformaldehyde for 2 hours and processed for imaging. Specimens were examined using an Olympus FV1000 confocal microscope. Images were obtained with identical settings with ×10 objective lens. To quantify GFP-positive cells, four regions of interest were randomly chosen per image at ×10 magnification. Within one region, area of GFP-positive cells and area of tissue in bright field were counted by software. The data are expressed as % of total area of GFP-positive in the region of AAV-CPP-treated tissues normalized by that of tissues treated by AAV2 alone.

### Animal experiments and immunohistochemistry

AAV2 (10^9^ particles/mouse) or AAV8 (10^8^ particles/mouse) was preincubated with Antp, TAT-HA2, or LAH4 (final concentration 10 μmol/l) for 30 minutes at 37 °C. The AAV or AAV-CPP complexes were intramuscularly injected into BALB/c mice. Fourteen days or 30 days after injection, mice muscles were excised, fixed, frozen, cryosectioned, and then mounted onto glass slides. Frozen sections were then fixed and rinsed with cold PBS. After blocking and permeabilization, the slides were washed by PBS and then incubated with mouse anti-GFP (Invitrogen), rat anti-mouse CD4 (Biolegend, San Diego, CA), or biotin anti-mouse CD8 (Biolegend), followed by secondary antibody, and counterstained with DAPI (Invitrogen). Fluorescence images were acquired by a Yokogawa spinning-disk confocal scanner system (Solamere Technology Group, Salt Lake City, UT) using a Nikon Eclipse Ti-E microscope. Illumination powers at 405, 491, 561, and 640 nm solid-state laser lines were provided by an acousto-optical tunable filter-controlled laser-merge system with 50 mW for each laser. All images were analyzed using Nikon NIS-Elements software. To quantify GFP-positive cells, four regions of interest were randomly chosen per image at ×10 magnification. These data were expressed as % of total nuclear area stained by GFP in the region. For toxicity, the frozen muscle sections were stained with hematoxylin and eosin. Histopathologic specimens were examined by light microscopy at ×20 magnification.

### Quantification of genome copies and cytokines

DNA of vectors was extracted using the QIAamp MinElute Virus Spin Kit (Qiagen, Valencia, CA) according to the manufacturer’s protocol. Quantitative PCR was performed on the Bio-Rad MyiQ real-time system using a pair of primers specific for the GFP transgene: 5′-GACATCATGAAGCCCCTTGAG-3′ (forward) and 5′-GGTGGTCGAAATTCAGATCAAC-3′ (reverse).

For *in vivo* biodistribution of AAV2, 14 days postinjection, organs from AAV2-injected and AAV2-CPP-injected mice were collected, homogenized, and purified using DNeasy Blood & Tissue kit (Qiagen). Quantitative polymerase chain reaction was then performed for quantification of GFP and endogenous mouse apolipoprotein B (Apob) in various organs. A primer set of Apob was used (forward primer 5′-CGTGGGCTCCAGCATTCTA-3′, reverse primer 5′-TCACCAGTCATTTCTGCCTTTG-3′). Apob DNA template was purchased from Bio-Rad (Hercules, CA).

For detection of cytokines in muscles, 14 days postinjection, total RNA was isolated from muscles using RNeasy Mini kit (Qiagen), and first-strand cDNA was synthesized using a QuantiTect Reverse Transcription kit (Qiagen). mRNA was detected using primer sets of IL-1b (forward primer 5′-GCAACTGTTCCTGAACTCAAC-3′, reverse primer 5′-ATCTTTTGGGGTCCGTCAACT-3′), IL-6 (forward primer 5′-TAGTCCTTCCTACCCCAATTTCC-3′, reverse primer 5′-TTGGTCCTTAGCCACTCCTTC-3′) and TNF-α (forward primer 5′-CTGAACTTCGGGGTGATCGG-3′, reverse primer 5′-GGCTTGTCACTCGAATTTTGA-3′). For an internal control, a primer set of GAPDH was used (forward primer 5′-AGGTCGGTGTGAACGGATTTG-3′, reverse primer 5′-TGTAGACCATGTAGTTGAGGT-3′).

### Statistical analysis

The results were expressed as means ± standard deviation. The significance of the difference in the means was determined by Student *t-*test.

## Figures and Tables

**Table 1:** Amino acid sequences of cell-permeable peptides used in this study

*Cell-permeable peptides*	*Peptide sequence*
Antp	RQIKIWFQNRRMKWKKC
TAT-HA2	CRRRQRRKKRGGDIMGEWGNEIFGAIAGFLG
LAH4	KKALLALALHHLAHLALHLALALKKAC
